# Supplementary material for: Low Levels of TRIM28-Interacting KRAB-ZNF Genes Associate with Cancer Stemness and Predict Poor Prognosis of Kidney Renal Clear Cell Carcinoma Patients
Source: Cancers (Basel). 2021 Sep 28;13(19):4835. doi: 10.3390/cancers13194835 (PMC8508054; doi:10.3390/cancers13194835)
Supplement: Supplementary file 1 [file cancers-13-04835-s001.zip › cancers-1383894-supplementary.pdf]

Supplementary Figures 1-8

Low levels of TRIM28-interacting KRAB-ZNF genes associate with cancer stemness and predict poor prognosis of Kidney Renal Clear Cell Carcinoma patients

Czerwinska Patrycja and Mackiewicz Andrzej Adam.

Figure S1.

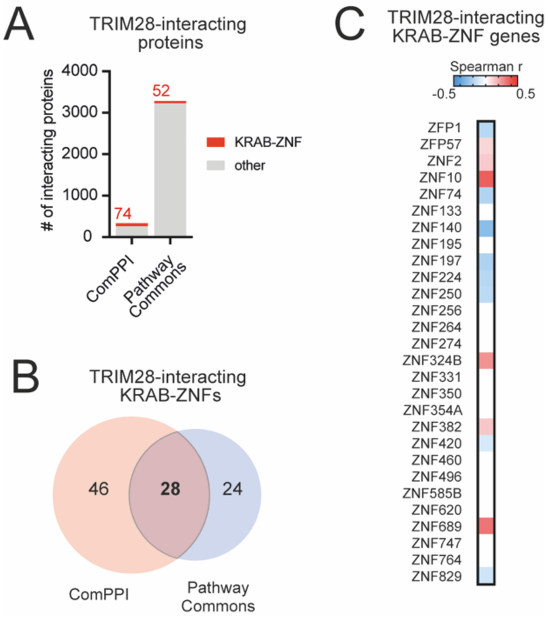

Figure S1. TRIM28-interacting KRAB-ZNF proteins.

A. The number of TRIM28-interacting proteins according to ComPPI and Pathway Commons databases. Red – the number of KRAB-ZNF proteins, grey – other proteins.
B. Venn's diagram of common KRAB-ZNFs that interact with TRIM28 according to both databases. 28 KRAB-ZNFs were detected as directly interacting with TRIM28 in both ComPPI and Pathway Commons.
C. Spearman correlation of KRAB-ZNFs expression and TRIM28 level in TCGA KIRC tumors. Blue and red denote negative and positive correlation, respectively. Only statistically significant correlations are shown ($p < 0.05$).

Figure S2.

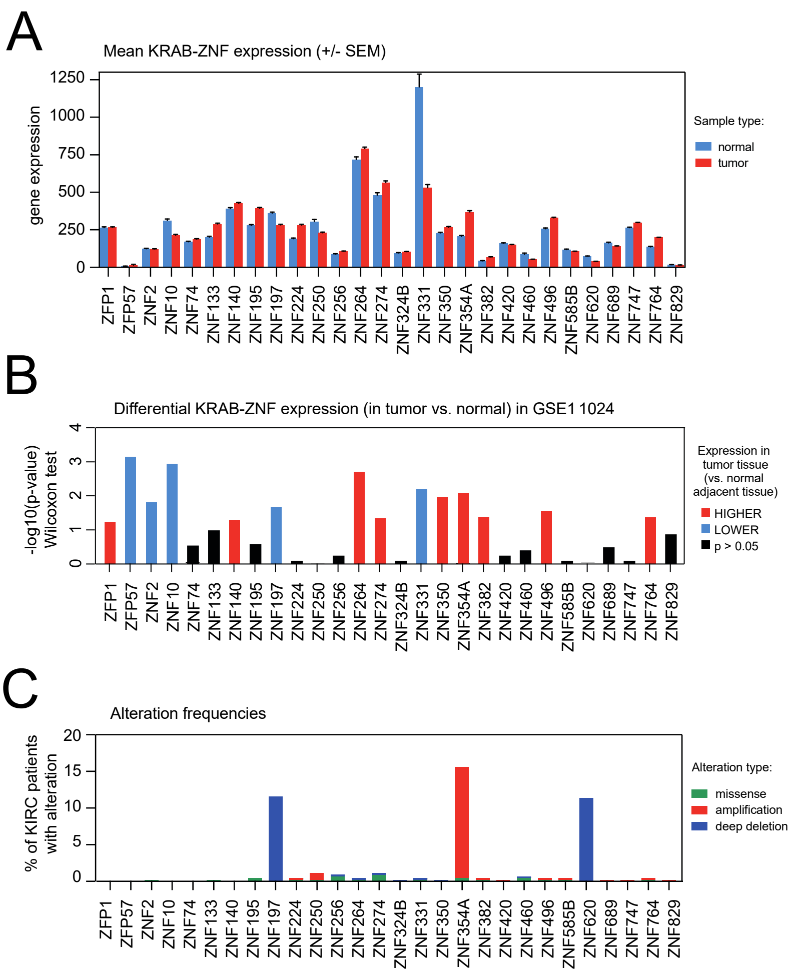

Figure S2. Gene expression and genetic alterations in TRIM28-interacting KRAB-ZNFs.

A. The mean expression (+/- SEM) of TRIM28-interacting KRAB-ZNFs in tumor tissues (red) and normal adjacent tissues (blue) based on TCGA KIRC data.
B. Differential expression of KRAB-ZNFs in kidney tumor vs. normal adjacent tissue based on GEO11024 dataset.
C. The frequencies of alterations in KRAB-ZNF proteins according to the cBioportal database. Green – missense mutations, red – amplifications, blue – deep deletions.

Figure S3.

Overall survival in KIRC

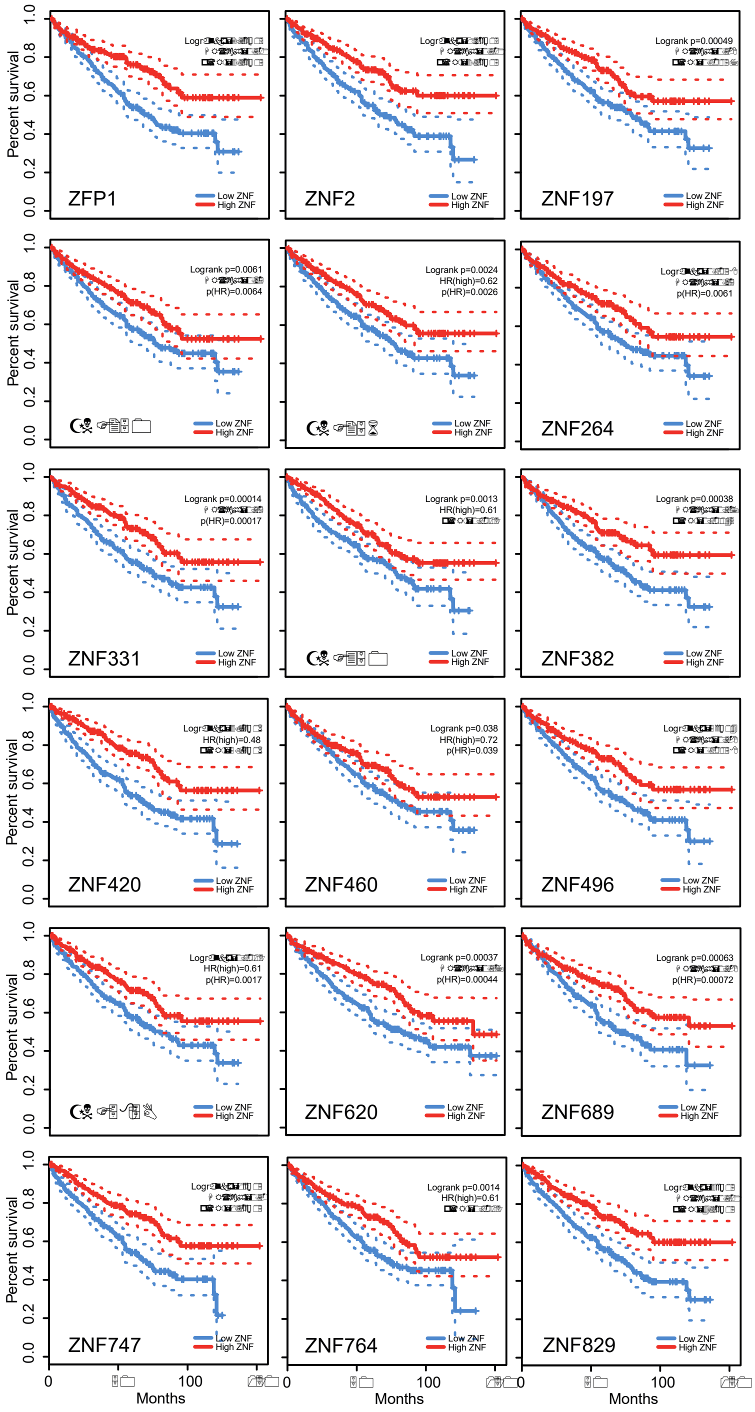

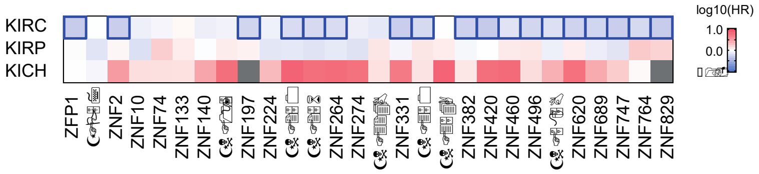

Figure S3. KRAB-ZNF association with the overall survival of TCGA KIRC patients.

A. Kaplan-Meier overall survival curves for KIRC patients separated into high- or low-expressing KRAB-ZNF cohorts (using the median expression as a cut-off). Red – high expression, blue – low expression of distinct KRAB-ZNFs.
B. The heatmap of the hazard ratios (log10(HR)) of death for TCGA kidney cancer patients with high expression of specific KRAB-ZNFs (using the median level as a cut-off). Red and blue denote higher or lower hazard ratios, respectively. KIRP – Kidney renal papillary cell carcinoma; KICH – kidney chromophobe cancer.

Figure S4.

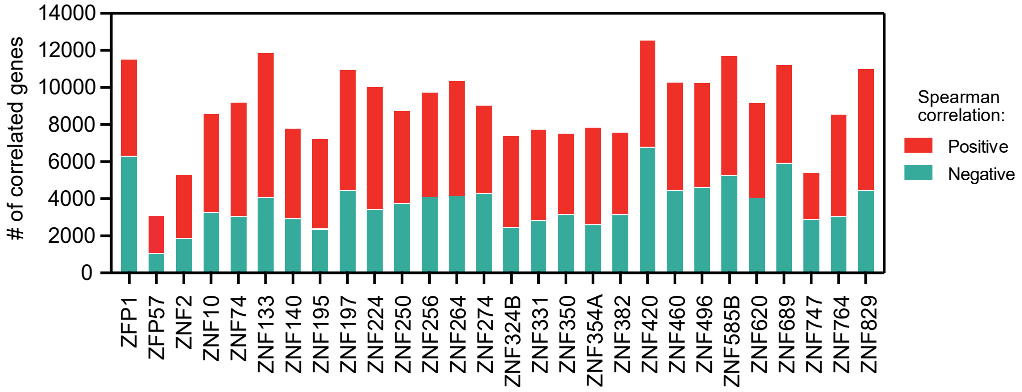

Figure S4. The transcriptome profiles associated with the expression of selected KRAB-ZNFs in TCGA KIRC tumors. The number of genes significantly correlated (adj. p-val < 0.01) with the expression of subsequent KRAB-ZNFs in KIRC is presented. Green and red denote negative and positive Spearman correlation, respectively.

Figure S5.

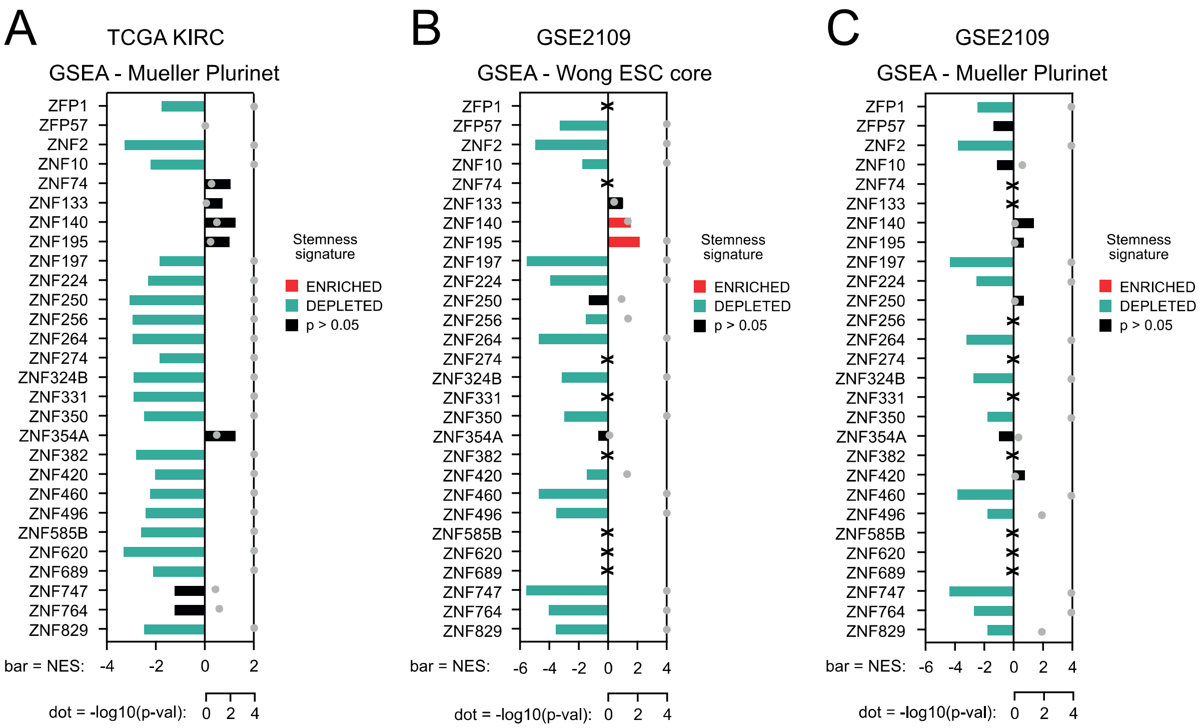

Figure S5. The transcriptome profiles of KRAB-ZNF^{HIGH} expressing patients are significantly enriched with stemness markers.

A. The GSEA using all significantly correlated genes (FDR < 0.01) to each of the tested KRAB-ZNFs in KIRC patients was performed with the stemness signature Mueller_Plurinet as a reference. Bar – the normalized enrichment score (NES). Grey dot – statistical significance (-log10FDR).

B-C. The GSEA using all significantly correlated genes (FDR < 0.01) to each of the tested KRAB-ZNFs in kidney tumors from the GSE2109 dataset was performed with the stemness signature (B) Wong_ESC_core or (C) Mueller_Plurinet as a reference. Bar – the normalized enrichment score (NES). Grey dot – statistical significance (-log10FDR). X – the gene set size threshold not passed.

Figure S6.

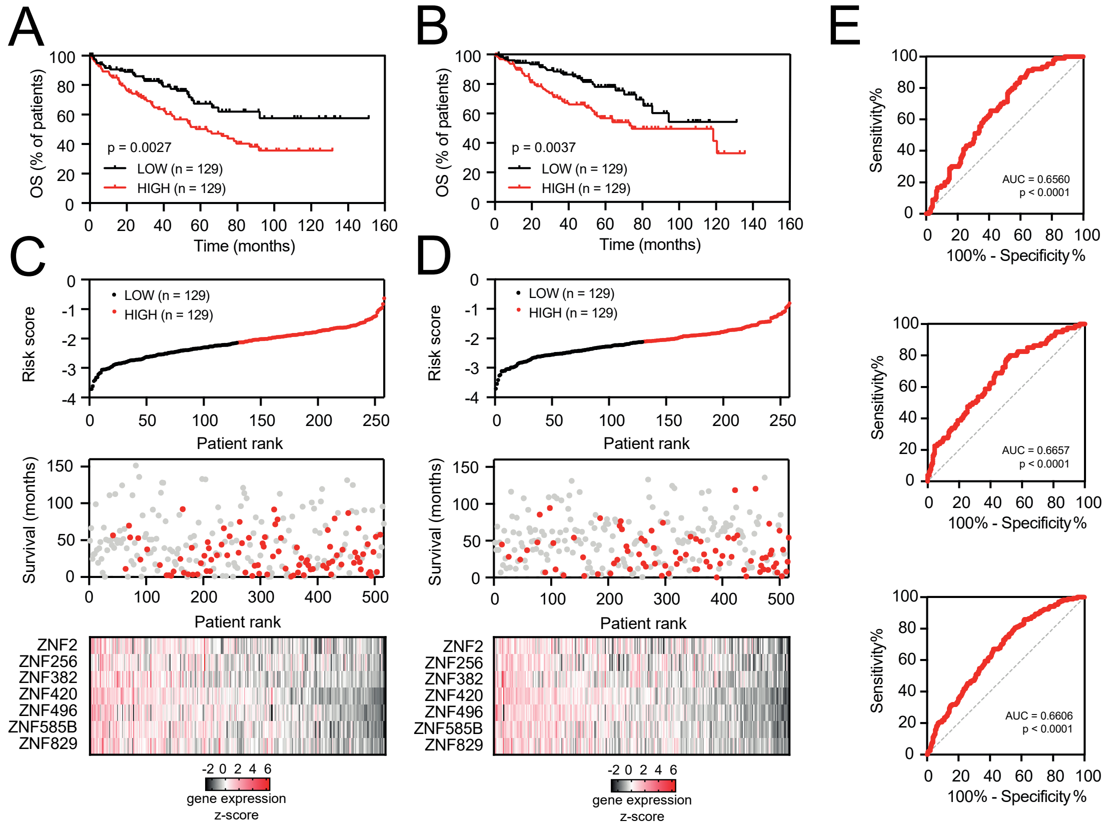

Figure S6. Development of the KRAB-ZNF-based gene signature for survival prediction in KIRC patients.

A-B. Kaplan-Meier survival curves for the discovery (A) and the validation (B) KIRC cohorts, respectively. All KIRC patients were randomly classified into either of the cohorts and stratified into high-risk and low-risk groups based on a median value of developed risk score. Red and black denote high- and low-risk patients, respectively.

C-D. The signature-based risk score distribution (upper panel: red – high risk, black – low risk), patients' survival status (middle panel: red – dead, grey – alive) and heatmap of seven KRAB-ZNFs expression profiles (lower panel: black – downregulated, red – upregulated expression) in the discovery (C) and the validation (D) KIRC cohort, respectively.

E. Diagnostic value of the KRAB-ZNF-based gene signature in the discovery (n = 258), the validation (n = 258), and the entire TCGA KIRC dataset (n = 516). The areas under the curve (AUC) were calculated for ROC curves, and sensitivity and specificity were calculated to assess the score performance.

Figure S7.

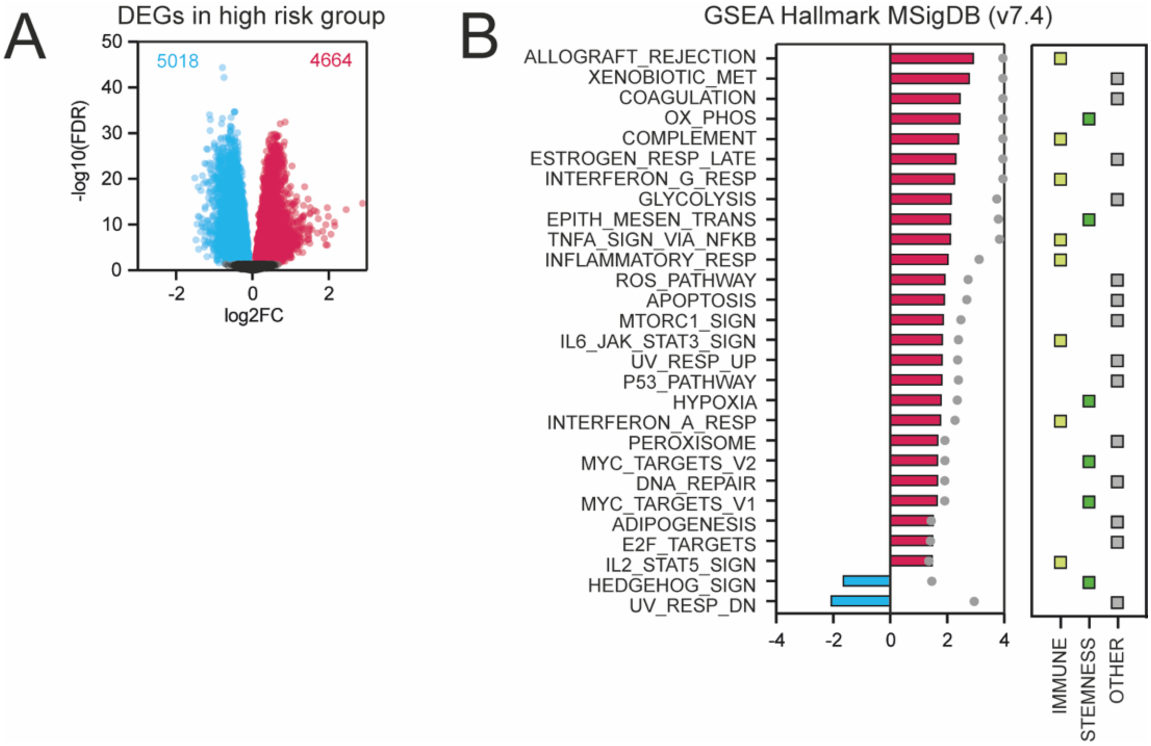

Figure S7. Transcriptome profiles of high-risk KIRC patients.

A. Volcano plot of differentially expressed genes between high risk and low-risk KIRC patients. Blue and magenta denote genes down- and up-regulated in high-risk KIRC patients, respectively. Cut-offs: p-value (t-test) < 0.05, FDR < 0.05.
B. All significantly differentially expressed genes (n = 9682) were sorted based on their log2FC value resulting in a pre-ranked gene list that was further used in a GSEA analysis with MSigDB Hallmark gene sets (v7.4) as a reference. Only biological processes with a nominal p-value < 0.05 and FDR < 5% are presented. Note significant up-regulation of immune-associated terms followed by significant up-regulation of stemness-associated terms in high-risk KIRC group. Bar – Normalized Enrichment Score (NES). Grey dot – statistical significance (-log10FDR).

Figure S8.

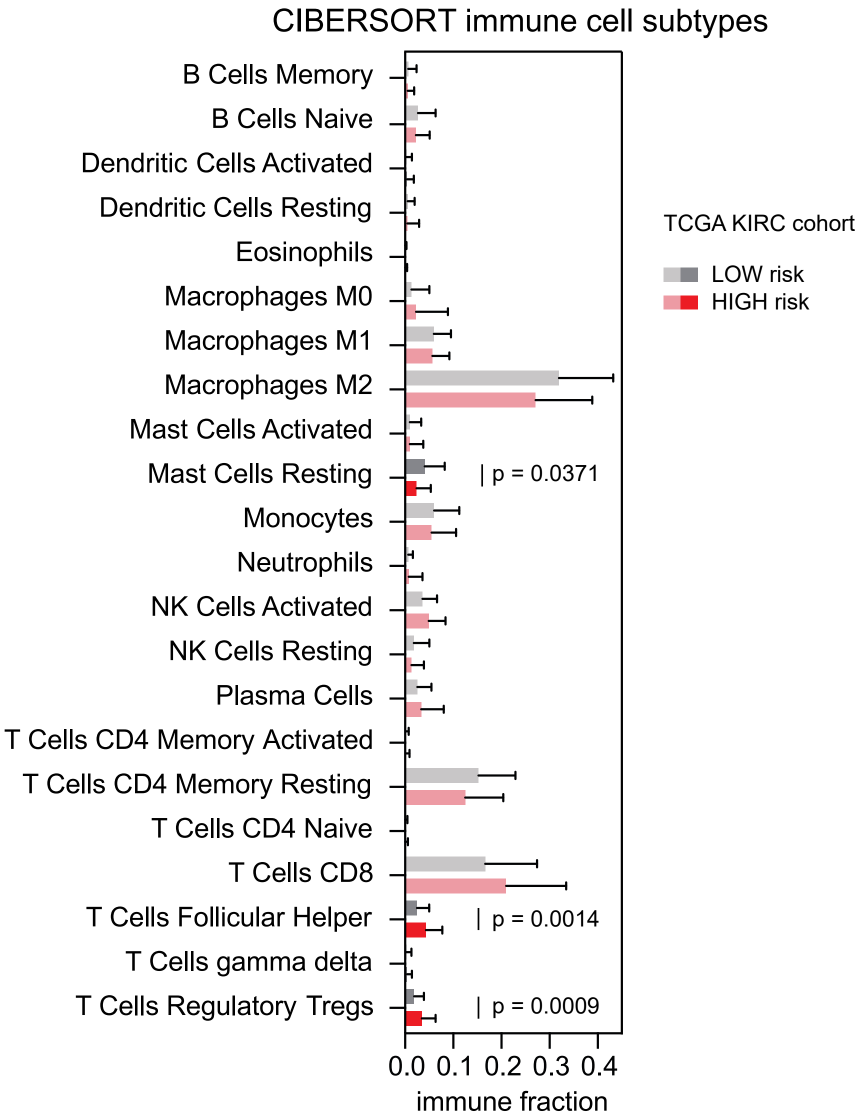

Figure S8. CIBERSORT-estimated immune cell fractions in high-risk and low-risk KIRC patients. Bar color intensity reflects statistical significance: light grey/red – $p > 0.05$; dark grey/red – $p < 0.05$.
